# *N*′-(4-Hydr­oxy-3-methoxy­benzyl­idene)acetohydrazide monohydrate

**DOI:** 10.1107/S1600536809037489

**Published:** 2009-09-26

**Authors:** Lu-Ping Lv, Wen-Bo Yu, Ying Tan, Yong-Zhao Zhang, Xian-Chao Hu

**Affiliations:** a Department of Chemical Engineering, Hangzhou Vocational and Technical College, Hangzhou 310018, People’s Republic of China; b Zhejiang Provincial Center for Disease Control and Prevention, Hangzhou 310051, People’s Republic of China; c Research Center of Analysis and Measurement, Zhejiang University of Technology, Hangzhou 310014, People’s Republic of China

## Abstract

In the title compound, C_10_H_12_N_2_O_3_·H_2_O, the Schiff base mol­ecule is approximately planar [within 0.189 (1) Å]. The inter­planar angle between the benzene and acetohydrazide planes is 8.50 (10)°. In the crystal, mol­ecules are linked into a three-dimensional network by strong and weak O—H⋯O and strong N—H⋯O hydrogen bonds. The hydr­oxy H atom deviates from the 4-hydr­oxy-3-methoxy­phenyl mean plane by 0.319 (2) Å, probably due to the involvement of this H atom in the O—H⋯O hydrogen bond. The weak O—H⋯O hydrogen bond is involved in a bifurcated hydrogen bond with *R*_1_^2^(4) motif. A weak C—H⋯π inter­action is also present.

## Related literature

For general background to Schiff bases, see: Cimerman *et al.* (1997 ▶); Offe *et al.* (1952 ▶); Richardson *et al.* (1988 ▶). For related structures, see: Li & Jian (2008 ▶); Tamboura *et al.* (2009 ▶). For hydrogen bonds, see: Desiraju & Steiner (1999 ▶); Etter *et al.* (1990 ▶).
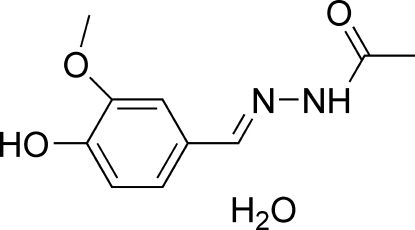


## Experimental

### 

#### Crystal data

C_10_H_12_N_2_O_3_·H_2_O
*M* *_r_* = 226.23
Orthorhombic, 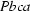

*a* = 7.892 (2) Å
*b* = 16.374 (5) Å
*c* = 18.334 (6) Å
*V* = 2369.3 (13) Å^3^
*Z* = 8
Mo *K*α radiation
μ = 0.10 mm^−1^
*T* = 223 K
0.24 × 0.20 × 0.18 mm

#### Data collection

Bruker SMART CCD area-detector diffractometer
Absorption correction: multi-scan (*SADABS*; Bruker, 2002 ▶) *T*_min_ = 0.977, *T*_max_ = 0.979
11089 measured reflections
2138 independent reflections
1484 reflections with *I* > 2σ(*I*)
*R*_int_ = 0.045

#### Refinement

*R*[*F* ^2^ > 2σ(*F* ^2^)] = 0.041
*wR*(*F* ^2^) = 0.115
*S* = 1.07
2138 reflections
159 parameters
1 restraint
H atoms treated by a mixture of independent and constrained refinement
Δρ_max_ = 0.14 e Å^−3^
Δρ_min_ = −0.19 e Å^−3^

### 

Data collection: *SMART* (Bruker, 2002 ▶); cell refinement: *SAINT* (Bruker, 2002 ▶); data reduction: *SAINT*; program(s) used to solve structure: *SHELXS97* (Sheldrick, 2008 ▶); program(s) used to refine structure: *SHELXL97* (Sheldrick, 2008 ▶); molecular graphics: *SHELXTL* (Sheldrick, 2008 ▶); software used to prepare material for publication: *SHELXTL*.

## Supplementary Material

Crystal structure: contains datablocks I, global. DOI: 10.1107/S1600536809037489/fb2165sup1.cif

Structure factors: contains datablocks I. DOI: 10.1107/S1600536809037489/fb2165Isup2.hkl

Additional supplementary materials:  crystallographic information; 3D view; checkCIF report

## Figures and Tables

**Table 1:** Hydrogen-bond geometry (Å, °)

*D*—H⋯*A*	*D*—H	H⋯*A*	*D*⋯*A*	*D*—H⋯*A*
O1—H1⋯O1*W*	0.93 (2)	1.69 (2)	2.614 (2)	170 (2)
O1*W*—H9*B*⋯O1^i^	0.87 (3)	2.19 (3)	2.899 (2)	139 (2)
O1*W*—H9*B*⋯O2^i^	0.87 (3)	2.27 (2)	3.0506 (19)	148 (2)
N2—H2⋯O3^ii^	0.837 (15)	2.023 (15)	2.851 (2)	169.6 (18)
O1*W*—H9*A*⋯O3^iii^	0.87 (2)	1.91 (2)	2.768 (2)	167 (2)
C10—H10*C*⋯*Cg*1^iv^	0.96	2.91	3.581 (3)	128

Symmetry codes: (i) 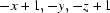; (ii) 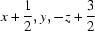; (iii) 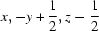; (iv) 
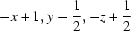
. *Cg*1 is the centroid of the C2–C7 ring.

**Fig. 1.**
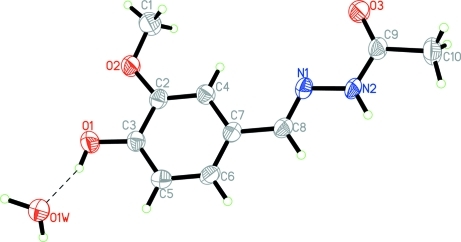
The asymmetric unit of the title structure. The displacement ellipsoids are drawn at the 40% probability level. The dashed lines indicate the hydrogen bonds.

**Fig. 2.**
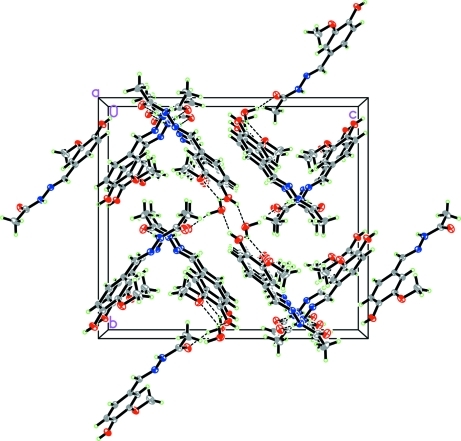
Molecular packing in the title compound. Hydrogen bonds are shown as dashed lines.

## supplementary crystallographic information

### Comment

Schiff bases have attracted much attention due to possibility of their analytical applications (Cimerman *et al.*, 1997). They are also important ligands, which have been reported to show mild bacteriostatic activity and to be potential oral iron-chelating drugs for genetic disorders such as thalassemia (Offe *et al.*, 1952; Richardson *et al.*, 1988). Metal complexes based on Schiff bases have received considerable attention because they can be utilized as model compounds with active centres in various complexes (Tamboura *et al.*, 2009). Here we report the crystal structure of  the title compound (Fig. 1).

In the Schiff base molecule, the acetohydrazide group is planar and it contains a dihedral angle equal 8.50 (10)° to the benzene ring. The molecule adopts the *trans* configuration with respect to the C═N bond. Bond lengths and angles are comparable to those observed for *N*'-[1-(4-methoxyphenyl)ethylidene]acetohydrazide (Li *et al.*, 2008).

In the crystal structure, the Schiff base and water molecules are linked into a three-dimensional network by strong and weak (Desiraju & Steiner, 1999) O—H···O and strong N—H···O hydrogen bonds (Tab. 1). The weak O—H···O hydrogen bond is involved in the bifurcated hydrogen bond with the motif R^2^_1_(4) (Etter *et al.*, 1990). Intermolecular C—H···π interactions are also present in the structure. It is of interest, that the atom H1 of the hydroxyl group deviates significantly from the mean plane of 4-hydroxy-3-methoxyphenyl (the atoms C1-C7//O1//O2) by 0.319 (2)Å. This feature can be explained by its involvement into the O1—H1···O1W hydrogen bond (Tab. 1).

### Experimental

4-Hydroxy-3-methoxybenzaldehyde (1.50 g, 0.01 mol) and acetohydrazide (0.74 g, 0.01 mol) were dissolved in methanol (20 ml) and stirred for 1.5 h at room temperature. The resulting solid was filtered off and recrystallized from ethanol to give the title compound in 88% yield. Colourless single crystals (0.8 × 0.6 × 0.5mm)  suitable for X-ray analysis were obtained by slow evaporation from ethanol solution at room temperature (m. p. 492–494 K).

### Refinement

All the hydrogen atoms could have been discerned in the difference electron density map, nevertheless, all the hydrogens attached to the carbon atoms were constrained in a riding motion approximation: C_aryl_-H = 0.93, C_methyl_-H = 0.96Å; U_iso_H_aryl_ = 1.2U_eq_C_aryl_, U_iso_H_methyl_=1.5U_eq_C_methyl_. The coordinates of the water hydrogens were freely refined with U_iso_H_Ow_=1.5U_eq_Ow. The N2-H2 distance was restrained to 0.87 (2) Å, U_iso_H2 =1.2U_eq_N2.

### Crystal data

**Table:**

C_10_H_12_N_2_O_3_·H_2_O	*D*_x_ = 1.268 Mg m^−^^3^
*M**_r_* = 226.23	Melting point = 492–494 K
Orthorhombic, *P**b**c**a*	Mo *K*α radiation, λ = 0.71073 Å
Hall symbol: -P 2ac 2ab	Cell parameters from 2085 reflections
*a* = 7.892 (2) Å	θ = 2.2–25.0°
*b* = 16.374 (5) Å	µ = 0.10 mm^−^^1^
*c* = 18.334 (6) Å	*T* = 223 K
*V* = 2369.3 (13) Å^3^	Block, colourless
*Z* = 8	0.24 × 0.20 × 0.18 mm
*F*(000) = 960	

### Data collection

**Table:**

Bruker SMART CCD area-detector diffractometer	2138 independent reflections
Radiation source: fine-focus sealed tube	1484 reflections with *I* > 2σ(*I*)
graphite	*R*_int_ = 0.045
φ and ω scans	θ_max_ = 25.4°, θ_min_ = 2.2°
Absorption correction: multi-scan (*SADABS*; Bruker, 2002)	*h* = −9→8
*T*_min_ = 0.977, *T*_max_ = 0.979	*k* = −19→19
11089 measured reflections	*l* = −21→21

### Refinement

**Table:**

Refinement on *F*^2^	Primary atom site location: structure-invariant direct methods
Least-squares matrix: full	Secondary atom site location: difference Fourier map
*R*[*F*^2^ > 2σ(*F*^2^)] = 0.041	Hydrogen site location: difference Fourier map
*wR*(*F*^2^) = 0.115	H atoms treated by a mixture of independent and constrained refinement
*S* = 1.07	*w* = 1/[σ^2^(*F*_o_^2^) + (0.0602*P*)^2^ + 0.0171*P*] where *P* = (*F*_o_^2^ + 2*F*_c_^2^)/3
2138 reflections	(Δ/σ)_max_ < 0.001
159 parameters	Δρ_max_ = 0.14 e Å^−^^3^
1 restraint	Δρ_min_ = −0.18 e Å^−^^3^
41 constraints	

### Special details

**Table:**

Geometry. All esds (except the esd in the dihedral angle between two l.s. planes) are estimated using the full covariance matrix. The cell esds are taken into account individually in the estimation of esds in distances, angles and torsion angles; correlations between esds in cell parameters are only used when they are defined by crystal symmetry. An approximate (isotropic) treatment of cell esds is used for estimating esds involving l.s. planes.
Refinement. Refinement of F^2^ against ALL reflections. The weighted R-factor wR and goodness of fit S are based on F^2^, conventional R-factors R are based on F, with F set to zero for negative F^2^. The threshold expression of F^2^ > 2sigma(F^2^) is used only for calculating R-factors(gt) etc. and is not relevant to the choice of reflections for refinement. R-factors based on F^2^ are statistically about twice as large as those based on F, and R- factors based on ALL data will be even larger.

### Fractional atomic coordinates and isotropic or equivalent isotropic displacement parameters (Å^2^)

**Table:**

	*x*	*y*	*z*	*U*_iso_*/*U*_eq_	
O2	0.44998 (15)	0.16677 (8)	0.62044 (7)	0.0551 (4)	
O1	0.58987 (17)	0.08238 (8)	0.51886 (7)	0.0548 (4)	
H1	0.661 (3)	0.0446 (14)	0.4961 (12)	0.082*	
N2	1.03345 (18)	0.42155 (9)	0.73839 (8)	0.0446 (4)	
H2	1.131 (2)	0.4257 (11)	0.7207 (10)	0.053*	
O3	0.85188 (16)	0.45567 (8)	0.82838 (7)	0.0593 (4)	
N1	0.92032 (17)	0.36650 (8)	0.70786 (7)	0.0414 (4)	
C7	0.8701 (2)	0.26735 (10)	0.61434 (9)	0.0400 (4)	
C3	0.6871 (2)	0.14216 (10)	0.54766 (9)	0.0404 (4)	
C4	0.7033 (2)	0.25087 (10)	0.63651 (9)	0.0415 (4)	
H4	0.6540	0.2817	0.6735	0.050*	
C2	0.6124 (2)	0.18899 (10)	0.60350 (9)	0.0399 (4)	
C9	0.9926 (2)	0.46154 (11)	0.79905 (10)	0.0471 (5)	
C8	0.9721 (2)	0.32982 (10)	0.65066 (9)	0.0427 (5)	
H8	1.0776	0.3432	0.6315	0.051*	
C5	0.8503 (2)	0.15935 (11)	0.52546 (9)	0.0453 (5)	
H5	0.8992	0.1293	0.4879	0.054*	
C6	0.9417 (2)	0.22132 (10)	0.55890 (9)	0.0452 (5)	
H6	1.0521	0.2321	0.5440	0.054*	
C10	1.1285 (3)	0.51521 (13)	0.83054 (11)	0.0679 (6)	
H10A	1.2178	0.5219	0.7955	0.102*	
H10B	1.1732	0.4904	0.8739	0.102*	
H10C	1.0815	0.5676	0.8423	0.102*	
C1	0.3699 (3)	0.20816 (14)	0.67877 (12)	0.0760 (7)	
H1A	0.2576	0.1868	0.6854	0.114*	
H1B	0.3636	0.2654	0.6679	0.114*	
H1C	0.4342	0.2003	0.7227	0.114*	
O1W	0.75904 (19)	−0.03511 (8)	0.45548 (8)	0.0579 (4)	
H9A	0.791 (3)	−0.0175 (14)	0.4128 (12)	0.087*	
H9B	0.676 (3)	−0.0698 (15)	0.4498 (13)	0.087*	

### Atomic displacement parameters (Å^2^)

**Table:**

	*U*^11^	*U*^22^	*U*^33^	*U*^12^	*U*^13^	*U*^23^
O2	0.0375 (8)	0.0614 (9)	0.0665 (9)	−0.0106 (6)	0.0067 (7)	−0.0210 (6)
O1	0.0473 (9)	0.0537 (8)	0.0635 (8)	−0.0095 (7)	0.0024 (7)	−0.0189 (6)
N2	0.0254 (8)	0.0517 (9)	0.0566 (10)	−0.0096 (7)	0.0013 (7)	−0.0094 (7)
O3	0.0361 (8)	0.0803 (10)	0.0615 (9)	−0.0070 (7)	0.0040 (7)	−0.0184 (7)
N1	0.0317 (9)	0.0435 (8)	0.0489 (9)	−0.0073 (6)	−0.0042 (7)	−0.0020 (7)
C7	0.0381 (11)	0.0419 (10)	0.0398 (9)	−0.0054 (8)	−0.0028 (8)	0.0050 (7)
C3	0.0413 (11)	0.0396 (9)	0.0402 (9)	−0.0029 (8)	−0.0045 (8)	0.0004 (8)
C4	0.0366 (11)	0.0447 (10)	0.0431 (9)	−0.0001 (8)	−0.0025 (8)	−0.0032 (7)
C2	0.0322 (10)	0.0421 (10)	0.0454 (10)	−0.0015 (8)	−0.0025 (8)	0.0005 (8)
C9	0.0324 (11)	0.0516 (11)	0.0571 (12)	−0.0013 (9)	−0.0033 (9)	−0.0094 (9)
C8	0.0339 (10)	0.0473 (10)	0.0469 (11)	−0.0060 (8)	0.0008 (8)	0.0020 (8)
C5	0.0478 (12)	0.0494 (11)	0.0387 (10)	−0.0036 (9)	0.0053 (8)	−0.0012 (8)
C6	0.0394 (11)	0.0502 (11)	0.0459 (10)	−0.0095 (8)	0.0068 (8)	0.0046 (8)
C10	0.0428 (13)	0.0720 (14)	0.0890 (16)	−0.0084 (11)	−0.0020 (11)	−0.0344 (12)
C1	0.0428 (13)	0.0937 (17)	0.0913 (16)	−0.0117 (12)	0.0174 (12)	−0.0392 (13)
O1W	0.0526 (10)	0.0591 (9)	0.0620 (8)	−0.0126 (7)	0.0080 (7)	−0.0117 (7)

### Geometric parameters (Å, °)

**Table:**

O2—C2	1.368 (2)	C4—H4	0.9300
O2—C1	1.415 (2)	C9—C10	1.502 (3)
O1—C3	1.351 (2)	C8—H8	0.9300
O1—H1	0.93 (2)	C5—C6	1.388 (2)
N2—C9	1.330 (2)	C5—H5	0.9300
N2—N1	1.3868 (19)	C6—H6	0.9300
N2—H2	0.837 (15)	C10—H10A	0.9600
O3—C9	1.238 (2)	C10—H10B	0.9600
N1—C8	1.276 (2)	C10—H10C	0.9600
C7—C6	1.386 (2)	C1—H1A	0.9600
C7—C4	1.403 (2)	C1—H1B	0.9600
C7—C8	1.462 (2)	C1—H1C	0.9600
C3—C5	1.380 (3)	O1W—H9A	0.87 (2)
C3—C2	1.408 (2)	O1W—H9B	0.87 (3)
C4—C2	1.381 (2)		
			
C2—O2—C1	117.56 (14)	N1—C8—H8	119.1
C3—O1—H1	108.4 (15)	C7—C8—H8	119.1
C9—N2—N1	120.09 (15)	C3—C5—C6	120.27 (16)
C9—N2—H2	120.5 (13)	C3—C5—H5	119.9
N1—N2—H2	119.1 (13)	C6—C5—H5	119.9
C8—N1—N2	115.58 (14)	C7—C6—C5	120.65 (16)
C6—C7—C4	119.35 (16)	C7—C6—H6	119.7
C6—C7—C8	119.34 (16)	C5—C6—H6	119.7
C4—C7—C8	121.25 (16)	C9—C10—H10A	109.5
O1—C3—C5	124.23 (16)	C9—C10—H10B	109.5
O1—C3—C2	116.17 (16)	H10A—C10—H10B	109.5
C5—C3—C2	119.60 (16)	C9—C10—H10C	109.5
C2—C4—C7	120.09 (16)	H10A—C10—H10C	109.5
C2—C4—H4	120.0	H10B—C10—H10C	109.5
C7—C4—H4	120.0	O2—C1—H1A	109.5
O2—C2—C4	125.62 (15)	O2—C1—H1B	109.5
O2—C2—C3	114.35 (14)	H1A—C1—H1B	109.5
C4—C2—C3	120.02 (16)	O2—C1—H1C	109.5
O3—C9—N2	122.85 (16)	H1A—C1—H1C	109.5
O3—C9—C10	121.28 (17)	H1B—C1—H1C	109.5
N2—C9—C10	115.87 (17)	H9A—O1W—H9B	109 (2)
N1—C8—C7	121.84 (16)		

### Hydrogen-bond geometry (Å, °)

**Table:**

*D*—H···*A*	*D*—H	H···*A*	*D*···*A*	*D*—H···*A*
O1—H1···O1W	0.93 (2)	1.69 (2)	2.614 (2)	170 (2)
O1W—H9B···O1^i^	0.87 (3)	2.19 (3)	2.899 (2)	139 (2)
O1W—H9B···O2^i^	0.87 (3)	2.27 (2)	3.0506 (19)	148 (2)
N2—H2···O3^ii^	0.84 (2)	2.02 (2)	2.851 (2)	170 (2)
O1W—H9A···O3^iii^	0.87 (2)	1.91 (2)	2.768 (2)	167 (2)
C10—H10C···Cg1^iv^	0.96	2.91	3.581 (3)	128

Symmetry codes: (i) −*x*+1, −*y*, −*z*+1; (ii) *x*+1/2, *y*, −*z*+3/2; (iii) *x*, −*y*+1/2, *z*−1/2; (iv) −*x*+1, *y*−1/2, −*z*+1/2.
